# Transfection of glycoprotein encoding mRNA for swift evaluation of N‐glycan engineering strategies

**DOI:** 10.1002/btpr.2990

**Published:** 2020-03-13

**Authors:** Nina Bydlinski, Michael T Coats, Daniel Maresch, Richard Strasser, Nicole Borth

**Affiliations:** ^1^ Department of Biotechnology BOKU University of Natural Resources and Life Sciences Vienna Austria; ^2^ Department of Chemistry BOKU University of Natural Resources and Life Sciences Vienna Austria; ^3^ Department of Applied Genetics and Cell Biology BOKU University of Natural Resources and Life Sciences Vienna Austria; ^4^ Austrian Centre of Industrial Biotechnology GmbH Graz Austria

## Abstract

N‐glycosylation is defined as a key quality attribute for the majority of complex biological therapeutics. Despite many N‐glycan engineering efforts, the demand to generate desired N‐glycan profiles that may vary for different proteins in a reproducible manner is still difficult to fulfill in many cases. Stable production of homogenous structures with a more demanding level of processing, for instance high degrees of branching and terminal sialylation, is particularly challenging. Among many other influential factors, the level of productivity can steer N‐glycosylation towards less mature N‐glycan structures. Recently, we introduced an mRNA transfection system capable of elucidating bottlenecks in the secretory pathway by stepwise increase of intracellular model protein mRNA load. Here, this system was applied to evaluate engineering strategies for enhanced N‐glycan processing. The tool proves to indeed be valuable for a quick assessment of engineering approaches on the cellular N‐glycosylation capacity at high productivity. The gene editing approaches tested include overexpression of key Golgi‐resident glycosyltransferases, partially coupled with multiple gene deletions. Changes in galactosylation, sialylation, and branching potential as well as N‐acetyllactosamine formation were evaluated.

## 1 INTRODUCTION

Adequate post‐translational processing is crucial for the efficacy of many protein therapeutics and the biopharmaceutical product´s requirements in this regard often determine the choice of expression system.^1^ Here, N‐glycosylation is considered as one of the most essential protein modifications. The fact that Chinese hamster ovary (CHO) cells produce N‐glycans that are human‐like promoted their status as the most commonly used production platform for therapeutic glycoproteins.^2^, ^3^

N‐glycosylation is closely monitored during cell line development as well as at the stage of manufacturing, as authorities only tolerate minimal batch‐to‐batch deviation to ensure stable performance and unchanged quality attributes.^4^, ^5^ Furthermore, several N‐glycosylation engineering strategies have been examined to enable the formation of specific N‐glycan patterns and enhance product efficacy—depending on the protein and the application in question. Monoclonal antibodies (mAb) of the immunoglobulin G (IgG) type have been most extensively studied in this regard, with the generation of defucosylated mAb with increased capacity for antibody‐dependent cellular cytotoxicity as a prominent example.^6^, ^7^, ^8^ Other glyco‐engineering approaches, especially those aiming for more extensively processed structures with high levels of branching and terminal sialylation, have yielded quite satisfactory results, but are far from reaching homogenous N‐glycan profiles for detailed structure–function relationship elucidation.^9^, ^10^

Above all, sialylation has been declared as a promising engineering target for many biological therapeutics.^11^ The degree of sialylation, the number of terminal N‐acetylneuraminic acid residues in N‐glycan structures, can significantly influence the circulating half‐life of some proteins by altering their affinity to cellular receptors,^12^ as well as their susceptibility to proteases.^13^ For enhanced sialylation, multiple studies have focused on erythropoietin (EPO),^10^, ^14^, ^15^, ^16^, ^17^, ^18^, ^19^ a heavily glycosylated protein with surface‐exposed and highly branched N‐glycan structures.^20^, ^21^

We previously demonstrated, that transfection of large quantities of glycoprotein encoding mRNA can push the N‐glycan processing machinery into limitations^22^: at high productivity of EPO‐Fc (fusion construct of EPO linked to an IgG derived Fc domain) the level of galactosylation and sialylation dropped. In addition, a severe reduction in N‐glycan branching with a gradual shift from tetra‐ and triantennary to biantennary N‐glycans was observed. In this report, we show that the mRNA transfection system is a useful tool to rapidly test N‐glycan engineering strategies that hold up to the challenge of high recombinant protein load.

## 2 RESULTS AND DISCUSSION

### 2.1 Generation of glyco‐engineered CHO‐K1 cell lines

**Figure 1:**
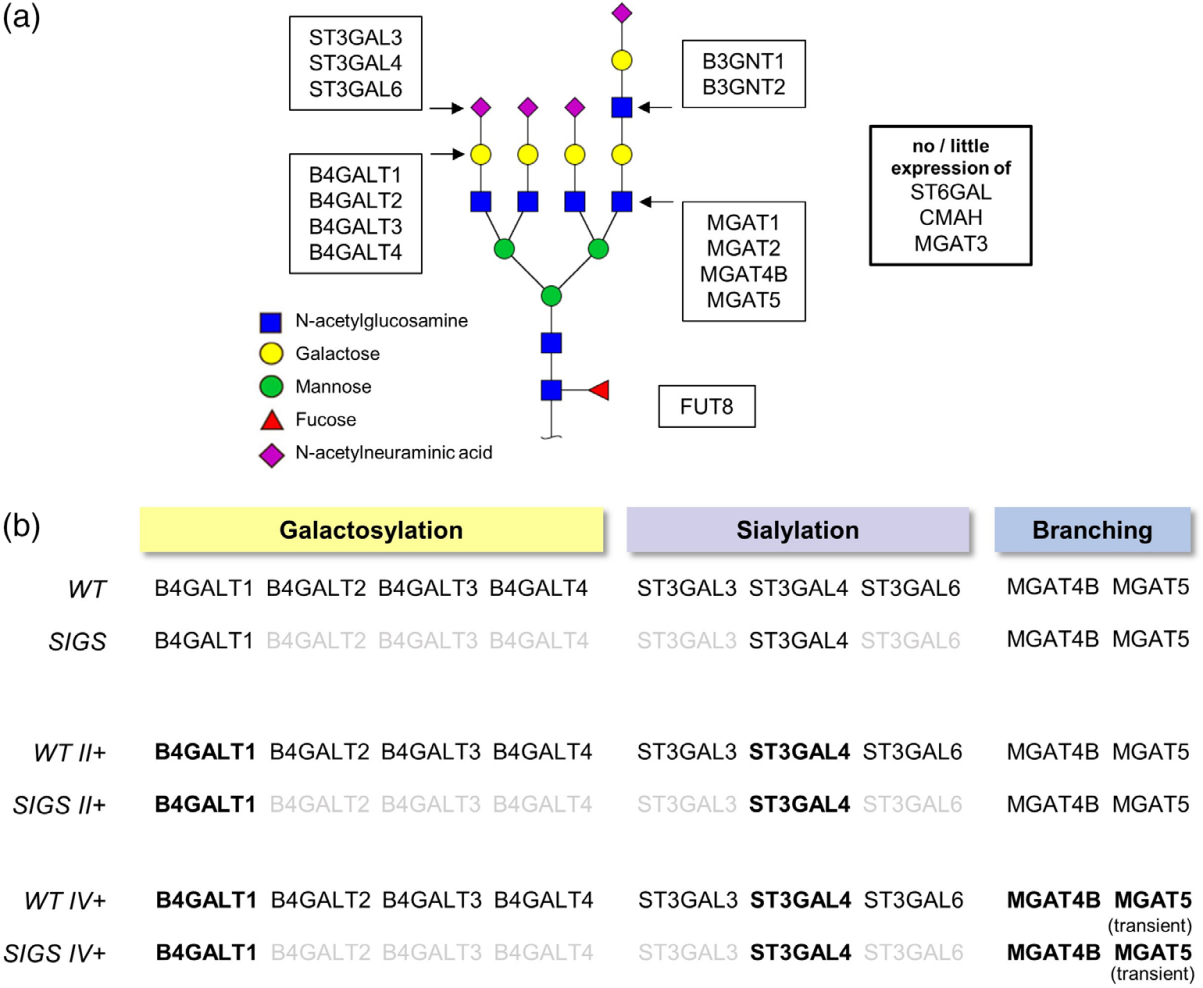
Overview of glycosyltransferase expression for all engineering strategies. (a) The transcriptome profile of CHO‐K1‐WT (WT) allows for full N‐glycan processing and includes several isoenzymes for certain modifications. (b) CHO‐K1‐SIGS (SIGS, single isoenzyme for galactosylation and sialylation) cell lines exhibit complete gene deletions of B4GALT2/3/4 as well as ST3GAL3/6 (marked in grey). After selection for stable integration of excess B4GALT1 and ST3GAL4 (enzyme overexpression marked in bold), single cell clones of WT II+ as well as SIGS II+ were characterized for key enzyme transcript levels by RT‐qPCR. Three clones each were selected for evaluation of their N‐glycosylation potential at high mRNA load. All clones were additionally screened in combination with transient overexpression of MGAT4B and MGAT5 (WT IV+ and SIGS IV+)

Presented approaches to enhance N‐glycosylation include sole overexpression of the key enzymes of galactosylation and sialylation in CHO‐K1, β‐1,4‐galactosyltransferase 1 (B4GALT1) and α‐2,3‐sialyltransferase 4 (ST3GAL4). In addition, a novel strategy to combine overexpression of selected glycosyltransferases with gene deletion of several glycosyltransferases that only contribute to N‐glycan processing to a small extent was evaluated.^23^, ^24^ Stable clones overexpressing both B4GALT1 and ST3GAL4 were generated with standard plasmids for protein expression in mammalian cells. They were modified by exchanging the CMV promoter cassettes with endogenous CHO‐K1 promoters P6‐RBS3 and P9‐FILA of moderate strength^25^ (for constructs see Data S1), to ensure increased transcript levels of B4GALT1 and ST3GAL4 while avoiding overload of the cells with Golgi‐resident transmembrane proteins. CHO‐K1 derived clones expressing B4GALT1 and ST3GAL4 as single isoenzymes for N‐glycan galactosylation and sialylation (expression pattern will be referred to as “SIGS”) were subjected to the same engineering strategy. Further, these engineered subclones were transiently transfected with the N‐acetylglucosaminyltransferases MGAT4B and MGAT5, to assess their effect on N‐glycan branching and their potential for further improvement of the strategies described. All glycosyltransferase transcript profiles evaluated in this report are summarized in Figure 1.

Material and methods used are briefly described in Data S1, including protocols for stable cell line generation and characterization, for the introduction of multiple genetic deletions by CRISPR/Cas9 and CRISPR/AsCpf1 using paired guide RNAs, for EPO‐Fc mRNA production and transfection and for glycopeptide analysis. These were presented in detail in Schmieder et al.,^26^ Bydlinski et al.,^22^ and Coats et al.,^22^ respectively.

### 2.2 Comparison of N‐glycan processing capacities at high EPO‐fc mRNA load

**Figure 2:**
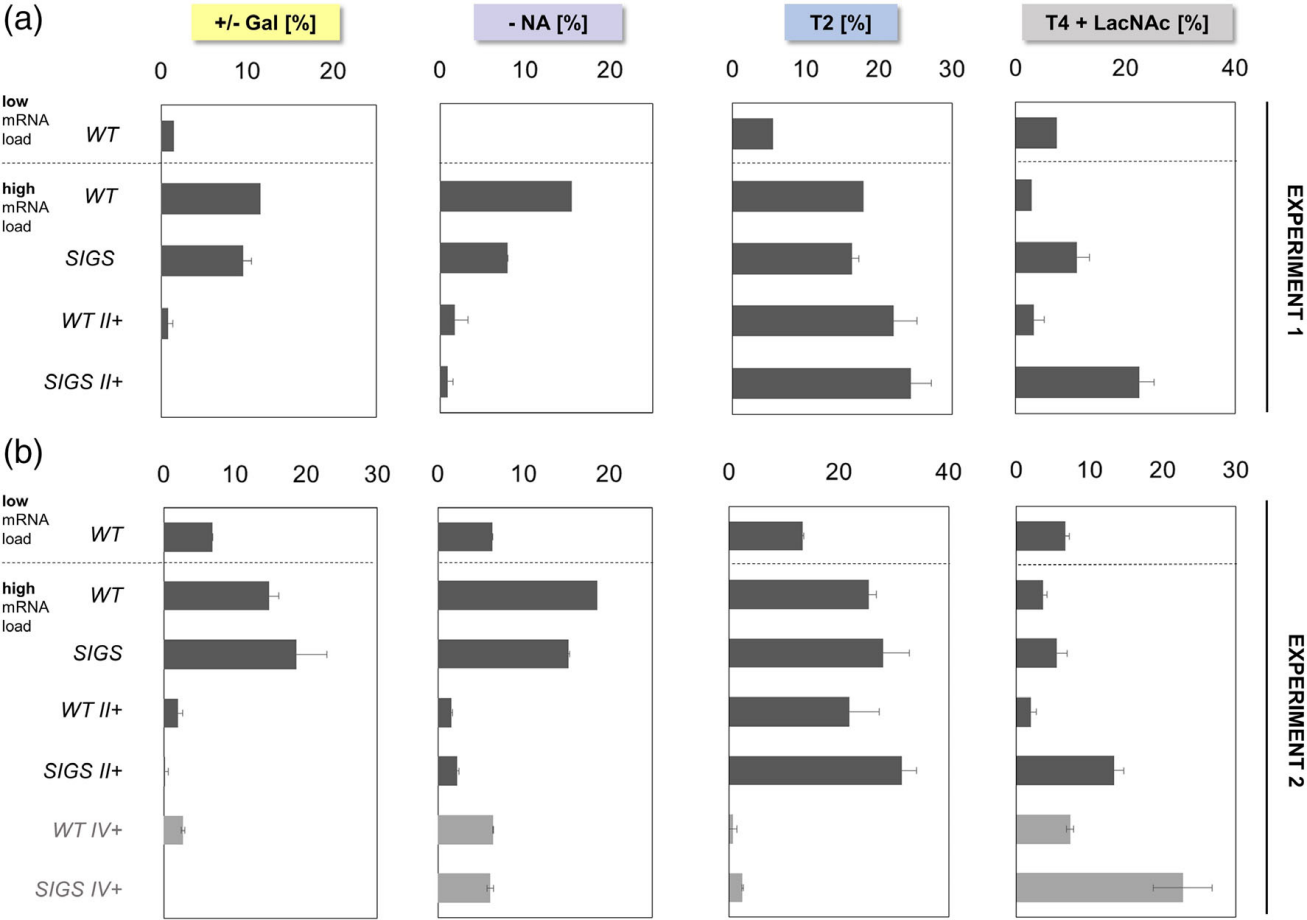
Degree of N‐glycan processing at Asn38 at high mRNA load for the individual engineering strategies. EPO‐Fc was purified from the supernatant by Protein A agarose beads and digested with trypsin. The resulting glycopeptides were analyzed by mass spectrometry (LC‐ESI‐MS). All detectable and identifiable N‐glycan structures were considered for relative quantification. The structures were grouped according to their level of galactosylation (incomplete galactosylation, “+/− Gal”), sialylation (no sialylation; “– NA”) and antennarity (biantennary glycans, “T2”). The sum of all tetra‐antennary T4 structures with at least one LacNAc unit (T4 extended, “T4 + LacNAc”) was also determined. Detailed data on the occurrence of individual N‐glycan structures is listed in Data S1. All stable engineering approaches (dark grey) were tested in two independent experiments. For WT II+ and SIGS II+, three individual clones were evaluated as biological replicates. Additionally, transient overexpression of MGAT4B and MGAT5 (light grey) was tested in all clones in a single replicate. Results are presented as average (±*SD*)

Transfections of high amounts (5 μg mRNA/ 6 × 10^5^ cells) compared to low amounts of EPO‐Fc mRNA (1 μg mRNA/ 6 × 10^5^ cells) showed a similar shift towards reduced galactosylation, sialylation and branching as observed previously.^22^ The changes in N‐glycosylation at Asn38 of EPO‐Fc, where the N‐glycan structure is most responsive to the increasing protein load, are depicted in Figure 2. First, the results verify that clones of the SIGS approach can still give rise to highly galactosylated and sialylated N‐glycans. Analysis of N‐glycosylation at Asn38 produced by SIGS cells at high mRNA load revealed small differences when compared to the N‐glycosylation capacity of regular CHO‐K1‐WT cells, with a trend towards less unsialylated structures for SIGS cell lines. When B4GALT1 and ST3GAL4 are stably overexpressed, the cell lines of the SIGS strategy, SIGS II+, show slightly improved processing compared to WT II+ with no or only traces of incompletely galactosylated N‐glycans.

Furthermore, all SIGS clones produced a higher fraction of tetra‐antennary (T4) structures that have N‐acetyllactosamine (LacNAc) units incorporated (elongation of galactose with N‐acetylglucosamine and another galactose moiety instead of capping with sialic acid). The occurrence of LacNAc units is further increased in SIGS clones with boosted B4GALT1, ST3GAL4 and MGAT4B/MGAT5 expression, SIGS IV+, where up to 25% of all structures are T4 N‐glycans carrying at least one LacNAc unit. The full extent of LacNAc modification could not be assessed in detail by the used mass spectrometry approach due to the occurrence of isobaric structures with different glycan compositions.

The most striking bottleneck elucidated during method establishment was the shift to biantennary (T2) glycan structures at high mRNA load, which could be counteracted by transiently introducing MGAT4B and MGAT5 in WT IV+ and SIGS IV+. Results of Experiment 2 show that clones of WT IV+ exhibit hardly any T2 structures (0–1.5%), compared to fractions of 16.8–31.6% for WT II+ cell lines. As SIGS cells exhibit higher levels of T4 + LacNAc structure, it is possible that here more T3 + LacNAc structures were misidentified as T4. We therefore refrained from direct comparison of T4 structures for the assessment of branching but only present the accumulation of T2 N‐glycans. These values are most likely less ambiguous, due to the substrate specificity of B3GNT2 that acts preferentially on β‐1,6‐branched antennas generated by MGAT5.^27^

Although relative differences between individual expression profiles are reproducible, values of N‐glycan structure distribution vary between experiments, consequently they were evaluated separately. This shift is most likely caused by differences in EPO‐Fc mRNA quality and purity, which can influence effective mRNA concentration. Evaluation of product titers for low load mRNA transfections of WT cultures showed higher EPO‐Fc yields for Experiment 2 than Experiment 1 (see Data S1), this could explain the overall shift to a lower processing level in Experiment 2. For high load mRNA transfections specific productivities above 15 pg/cell/day were determined (calculated for a 16–18 h time frame, but most product accumulation occurs within the first hours), previously we recorded similarly unfavorable effects on N‐glycosylation for all EPO‐Fc mRNA transfections that achieve 10 pg/cell/day or more.^22^ In coherence with the observations during initial method establishment, transfections of high EPO‐Fc mRNA load often resulted in reduced growth and a drop of cell culture viability, detailed data is available in Coats et al.^22^

The removal of nonessential Golgi glycosyltransferases for N‐glycosylation has potentially led to a shift towards increased LacNAc incorporation. This N‐glycan modification is initiated by N‐acetylglucosamine transfer onto galactose catalyzed by β‐1,3‐N‐acetylglucosaminyltransferases B3GNT1/2, with B3GNT2 being reported to be the dominant isoenzyme.^23^ At most processing steps in the Golgi, N‐glycans are potential substrates for multiple enzymes at a time and changes in glycosyltransferase expression levels have previously been described to shift the equilibrium of possible further modifications and therefore impact the resulting N‐glycan profiles.^19^, ^28^, ^29^ Competition between ST3GAL and B3GNT has been observed previously for interferon γ N‐glycan processing.^9^

LacNAc synthesis is rather poorly characterized for CHO cells, but overall this N‐glycan modification is regarded as undesirable as it increases N‐glycan heterogeneity. There is little elucidation of its structure–function relationship, for EPO it has been shown to negatively impact its circulating half‐life if multiple repeats are incorporated.^30^ Nevertheless, the combined results do suggest that CHO‐K1‐SIGS in general produce more highly processed N‐glycans at high mRNA load.

## 3 CONCLUSION

In this report, we show that the mock high productivity scenario based on flooding the cells with large amounts of product transcript, can elucidate bottlenecks in N‐glycan processing of recombinant proteins and verify the suitability of engineering strategies to overcome these limitations. Here, in this short interval set‐up in rich medium, the data indicate that in unmodified CHO‐K1‐WT cells the glycosyltransferase availability is indeed a limiting factor: boosting expression of key players could counteract reduced N‐glycan processing. Since Golgi nucleotide sugar concentrations still allowed for increased maturation levels in modified cell lines, supply of sugar building blocks is less likely to be a detrimental factor. However, this may change in systems with long‐term high‐productivity such as in production cell lines. Decreased growth rates often observed for high mRNA load transfections points towards insufficient supply of energy and/or specific metabolites to keep up with high protein production while maintaining normal growth behavior. Whether or not the clones that were subjected to glyco‐gene engineering would also behave superior when used as hosts for the generation of stable producer remains to be shown. Their N‐glycosylation capacity could be further optimized by combinatorial approaches targeting activated nucleotide sugar precursor synthesis,^17^ transport into the Golgi^31^ or by media supplementation.^32^, ^33^, ^34^

We propose that mimicking high productivity by mRNA transfections could also be used to similarly evaluate engineering targets in other processing steps along the secretory pathway, such as folding, proteolytic cleavage, O‐glycosylation or secretion rate. Since this mRNA system allows for testing numerous strategies upfront, at a very early stage of conception, the time‐consuming process of generating stable cell lines for difficult‐to‐express proteins could be avoided during initial screening.

The unexpected changes in LacNAc formation recall the fact that glycosyltransferases of the Golgi rely on a well‐orchestrated system with many direct interactions reported^35^, ^36^, ^37^, ^38^ and probably many mechanisms of organization not yet identified that enable high processing efficiency at low protein abundance. This network is prone to perturbation by severe changes of enzyme concentration and/or isoenzyme removal, resulting in alterations in N‐glycosylation patterns that are not fully predictable and where consequences might differ for individual proteins. In the future, with new therapeutic modalities moving forward, more sophisticated glycosylation engineering strategies, which for instance rely on precisely defined ratios of processing enzymes, will have to be developed to overcome current limitations. The potential of such strategies could be rapidly assessed with the method described here, rendering screening for protein‐customized optimization approaches more realistic.

## CONFLICT OF INTEREST

The authors declare no financial or commercial conflict of interest.

## NOTATION

CHO cells: Chinese hamster ovary cells
CRISPR/Cas9: clustered regularly interspaced short palindromic repeat editing system based on Cas9 endonuclease
CRISPR/AsCpf1: CRISPR system based on AsCpf1 endonuclease
mAb: monoclonal antibody
IgG: immunoglobulin G
EPO: erythropoietin
CMV promoter: cytomegalovirus promoter
Gal: galactose
NA: N‐acetylneuraminic acid
B4GALT: β‐1,4‐galactosyltransferase
ST3GAL: α‐2,3‐sialyltransferase
MGAT: N‐acetylglucosaminyltransferase
LacNAc: N‐acetyllactosamine
B3GNT: β‐1,3‐N‐acetylglucosaminyltransferase
T2 glycans: biantennary glycans
T4 glycans: tetra‐antennary glycans

## Supporting information


**Data S1** Supporting information.
