# Supplementary material for: Transfection of glycoprotein encoding mRNA for swift evaluation of N‐glycan engineering strategies
Source: Biotechnol Prog. 2020 Mar 13;36(4):e2990. doi: 10.1002/btpr.2990 (PMC7507192; doi:10.1002/btpr.2990)
Supplement: Supplementary file 1 — Data S1 Supporting information. [file BTPR-36-e2990-s001.docx]

**Supplementary Material**

**Material and Methods**

*Cell culture and transfection*

CHO-K1 cells were cultivated in CD-CHO medium (Thermo Fisher Scientific) supplemented with 8 mM L-Glutamine (Sigma Aldrich) and 0.2% Anti-Clumping Agent (Thermo Fisher Scientific), at 37°C, 7% CO_2_.

Transfections for plasmid delivery of Cas9/AsCpf1 constructs for gene deletions or glycosyltransferase encoding vectors were performed with the NEON® Nucleofection system (Thermo Fisher Scientific) and the NEON® 100 µl reaction kit. Routinely, 15- 20 µg DNA are transfected into 5 x 10^6^ cells by a single 20 ms pulse of 1700 V.

*Introducing genomic deletions of glycosyltransferases*

Full genomic deletions were introduced using CRISPR/Cas9 and a paired gRNA approach according to Bauer et al. 2015, only ST3GAL3 deletions were generated with CRISPR/AsCpf1 with a comparable protocol. After subcloning by limited dilution and clone expansion, screening for correct editing was done by non-deletion and deletion PCRs on crude cell lysates. Selected clones were reanalysed for complete transcript loss by RT-qPCR. RNA was isolated using the Direct-zol RNA MicroPrep kit (Zymo), cDNA was transcribed with the High-Capacity cDNA Reverse Transcription kit (Thermo Fisher Scientific) and qPCR reactions were in quadruplicates run on a Rotor-Gene qPCR cycler (Qiagen) using the SensiFAST SYBR® Hi-ROX kit (Bioline). Transcript levels were normalized to levels of *gapdh* mRNA and fold changes in target gene expression relative to CHO-K1 reference material were determined with the 2^-∆∆CT^ method. Detailed protocols are found in Schmieder et al., 2017 and Bydlinski et al., 2018.

gRNA sequences for gene deletion and primers used for clone characterization are listed in Table S1 and S2.

*Generation of clonal cell lines with glycosyltransferase overexpression*

B4GALT1 and ST3GAL4 were overexpressed in CHO-K1 (WT) cells as well as in a CHO-K1 derived clonal cell lines devoid of B4GALT2/3/4 and ST3GAL3/6 (SIGS). Stable pools were generated by selection with G418 (Invivogen, 700 µg/ml) and Hygromycin B gold (Invivogen, 300 µg/ml) for at least three weeks post transfection. Subcloning was performed by limited dilution and three clones of each were selected for subsequent experiments based on transcript levels of B4GALT1 and ST3GAL4. They were further cultivated with both selective agents, medium free of G418 and Hygromycin B was used during experiments for N-glycosylation potential determination. MGAT4B and MGAT5 were only overexpressed transiently during protein expression. Here, respective plasmids were transfected 48 h prior to start of protein production. All vectors for glycosyltransferase overexpression are listed in Table S3.

*EPO-Fc mRNA production, EPO-Fc expression and purification*

Precursor EPO-Fc mRNA was produced from the linearized, template vector pCI-neo_EPO-Fc (T7 promoter) using the AmpliScribe T7-Flash in-vitro transcription kit and purified with the MegaClear RNA purification kit (Thermo Fisher Scientific). The precursor-mRNA was capped and tailed with the ScriptCap m7G-capping kit (Cellscript, USA) and A-Plus Poly(A) tailing kit (Cellscript) and again purified. Quantity and quality were analyzed with NanoDrop One (Thermo Fisher Scientific) and BioAnalyzer (Agilent, USA) with the RNA Nano kit. More detailed protocols are found in Coats et al, 2019.

EPO-Fc mRNA was transfected with the NEON® Nucleofection system (Thermo Fisher Scientific) and the NEON® 10 µl reaction kit. Low load of mRNA refers to 1 µg EPO-Fc mRNA / 5 x 10^5^ cells, high load settings are 5 µg EPO-Fc mRNA / 5 x 10^5^ cells. The cells were electroporated with three 10 ms pulses at 1750 V, for each replicate 3 transfections were pooled and seeded into 6 ml media. Supernatant was harvested 16 – 18 h post transfection, filtered (0,22 µm) and EPO-Fc was purified using Protein A Agarose beads (Thermo Scientific).

*Site specific N-Glycosylation analysis by mass spectrometry*

Detailed protocols and information on material, equipment and the respective settings are decribed in Bydlinski et al., 2018. Briefly, EPO-Fc samples were run on an SDS-PAGE, followed by in-gel digestion with trypsin and labelling of peptides. LC-ESI-MS (Dionex Ultimate 3000 system directly linked to a Q-TOF instrument (maXis 4G ETD, Bruker) equipped with the standard ESI source) was performed and data obtained for glycopeptide HCSLNE*NIT*VPDTK, containing the reporter site Asn38, was quantified. For relative quantification of the different glycoforms, peak areas of EICs (extracted ion chromatograms) of the first four isotopic peaks of all glycopeptide derived signals were considered.

**Table S1** gRNA constructs and primers for clone characterization

| **gRNA constructs for genomic deletions** | |  |  |
| --- | --- | --- | --- |
| Name | Sequence (5'-3') | CRISPR system |  |
| B4GALT1_sg1 | TGATTGGCCACACTAGCGCT | Cas9 |  |
| B4GALT1_sg2 | AATCCGGTCAAACCTACCAG | Cas9 |  |
| B4GALT2_sg1 | TATGCGTGTATAGCGTTGTA | Cas9 |  |
| B4GALT2_sg2 | TCACGCCCAGATGTCCGGAT | Cas9 |  |
| B4GALT3_sg1 | GTCTCTGCTGTACAGGCGAC | Cas9 |  |
| B4GALT3_sg2 | AGAGGACCAGGCCTAGCGGG | Cas9 |  |
| B4GALT4_sg1 | GAAGTTCTCAAGTCCGCAAG | Cas9 |  |
| B4GALT4_sg2 | CCAGCTAGAATGCTTTACGC | Cas9 |  |
| ST3GAL3_up | ACAAACTGACCCGGTCACTCCGA | AsCpf1 |  |
| ST3GAL3_down | CATTCAGCGGGACACATCATCAG | AsCpf1 |  |
| ST3GAL6_sg1 | CACAGACATAATCGTTACTG | Cas9 |  |
| ST3GAL6_sg2 | GTGGCTCAATGGTGTTACGG | Cas9 |  |
|  |  |  |  |
| **Non-deletion PCR primers for screening** | |  |  |
| Name | Sequence (5'-3') | T anneal (°C) | Amplicon size (bp) |
| ND_B4GALT2_fw | GACCTGAATGCCTAAACCTGC | 60 | 650 |
| ND_B4GALT2_rev | TGACAGCCACAAGGAAGTGC |  |  |
| ND_B4GALT3_fw 4 | CCCACTAGGTTGCAGAAAGGT | 60 | 790 |
| ND_B4GALT3_rev 4 | AGGTAACAACCACCAGCAGTC |  |  |
| ND_B4GALT4_fw | CCCATTGCTGATGGATCATC | 60 | 540 |
| ND_B4GALT4_rev | GGCTATCAAGGCACTTCAC |  |  |
| ND_ST3GAL3_fw | GGGACGAGCGCAGGAAAGTC | 58 | 710 |
| ND_ST3GAL3_rev | GCTAGGTGGTGCTTGTTGACC |  |  |
| ND_ST3GAL6_fw | TATACGATGAACCTGTTCGCC | 58 | 1045 |
| ND_ST3GAL6_rev | CTCATTACGTCTCACTCAGCTAC |  |  |
|  |  |  |  |
| **Deletion PCR primers for screening** | |  |  |
| Name | Sequence (5'-3') | T anneal (°C) | Amplicon size (bp) |
| D_B4GALT2_fw | GACCTGAATGCCTAAACCTGC | 60 | 570 |
| D_B4GALT2_rev | GTCACCTCTAGGAACCAACCC |  |  |
| D_B4GALT3_fw | CCCACTAGGTTGCAGAAAGGT | 60 | 1050 |
| D_B4GALT3_rev | GTAGCCTGGGATGTAGAAAGC |  |  |
| D_B4GALT4_fw | AGTAAGCATTTCCAGGCAGC | 60 | 550 |
| D_B4GALT4_rev | GGCTATCAAGGCACTTCAC |  |  |
| D_ST3GAL3_fw | GGGACGAGCGCAGGAAAGTC | 58 | 485 |
| D_ST3GAL3_rev | ATGGTCACTGCCACACTGCC |  |  |
| D_ST3GAL6_fw | TATACGATGAACCTGTTCGCC | 58 | 900 |
| D_ST3GAL6_rev | CGCACACAATACACAGAATGC |  |  |

**Table S2** qPCR primer for analysis of glycosyltransferase expression level

| Name | Sequence (5'-3') | T anneal (°C) |
| --- | --- | --- |
| qPCR_GAPDH_fw | AACTTTGGCATTGTGGAAGG | 60 |
| qPCR_GAPDH_rev | ACACGTTGGGGGTAGGAACA |  |
| qPCR_B4GALT1_fw | GCCATCATTATCCCATTTCGC | 60 |
| qPCR_B4GALT1_rev | TCGATTGAACATGGTGTCTCC |  |
| qPCR_B4GALT2_fw | AAACATAACGAGCCCAACCC | 60 |
| qPCR_B4GALT2_rev | TCGCCCGATGTCCACTGTG |  |
| qPCR_B4GALT3_fw | GAAGATGAATGGCTTCCCCAA | 60 |
| qPCR_B4GALT3_rev | CCTCTGTGCTTCACCATCTTA |  |
| qPCR_B4GALT4_fw | AACTCTGGTCCATGATGCAA | 60 |
| qPCR_B4GALT4_rev | TGAGGTCCGGCTCAAAAATG |  |
| qPCR_ST3GAL3_fw | TTGACTCCGCTGGACAAACA | 60 |
| qPCR_ST3GAL3_rev | TAGCTCGGCAGGCAGTTTAG |  |
| qPCR_ST3GAL4_fw | ATGAGTTGCCCTTTGGGACC | 60 |
| qPCR_ST3GAL4_rev | CCACAACACAACGACGACAC |  |
| qPCR_ST3GAL6_fw | ATGTCCAAAACAGCCCCCTT | 60 |
| qPCR_ST3GAL6_rev | AGCTGCACACAGAAATGGG |  |

**Table S3** Glycosyltransferase encoding plasmids for overexpression

| glycosyltransferase | vector backbone | promoter | promoter strength | selective marker |
| --- | --- | --- | --- | --- |
| B4GALT1 | pcDNA3.1 | P9-FILA* | moderate | neomycin / G418 |
| ST3GAL4 | pNL2.2 | P6-RBS3* | moderate | hygromycin B |
| MGAT4B | pcDNA3.1 | CMV | strong | neomycin / G418 |
| MGAT5 | pcDNA3.1 | CMV | strong | neomycin / G418 |
|  |  |  |  |  |
| * endogenous promoter P9-FILA (filamin A) and P6-RBS3 (40s ribosomal protein S3a-like protein) were characterized for recombinant protein expression and promoter activity was determined in reference to CMV and SV40 promoter strength, additional information is found in Nguyen et al., 2019 | | | | |

**Supplementary data 1** N-Gycosylation at Asn38 of EPO-Fc (relative occurrence of structures in %) – Experiment 1

**Supplementary data 2** N-Gycosylation at Asn38 of EPO-Fc (relative occurrence of structures in %) – Experiment 2, part A

**Supplementary data 2 ctd.** N-Gycosylation at Asn38 of EPO-Fc (relative occurrence of structures in %) – Experiment 2, part B

**Supplementary data 3** Calculated specific productivity qP of EPO-Fc for WT cell lines in low (1 µg mRNA/ 6 x105 cells) and high amount (5 µg mRNA/ 6 x105 cells) mRNA transfections.

|  |  | **qP [pg/cell/day]** |
| --- | --- | --- |
| Experiment 1 - WT | 1 µg R1 | 3,9 |
|  | 1 µg R2* | 3,8 |
|  | 5 µg R1 | 15,5 |
|  | 5 µg R2* | 17,3 |
| Experiment 2 - WT | 1 µg R1 | 7,2 |
|  | 1 µg R2 | 7,4 |
|  | 5 µg R1 | 15,3 |
|  | 5 µg R2 | 17,0 |

* Replicates 2 of Experiment 1 were not subjected to N-glycan analysis
